# Strengthening district-based health reporting through the district health management information software system: the Ugandan experience

**DOI:** 10.1186/1472-6947-14-40

**Published:** 2014-05-13

**Authors:** Vincent Micheal Kiberu, Joseph KB Matovu, Fredrick Makumbi, Carol Kyozira, Eddie Mukooyo, Rhoda K Wanyenze

**Affiliations:** 1 Makerere University School of Public Health-U.S. Centers for Diseases Control and Prevention (MakSPH-CDC) Fellowship Program, P.O. Box 7072 Kampala, Uganda; 2 Department of Biostatistics and Epidemiology, Makerere University School of Public Health, Kampala, Uganda; 3 Ministry of Health, Resource Center, Kampala, Uganda; 4 Department of Disease Control and Environmental Health, Makerere University School of Public Health, Kampala, Uganda

**Keywords:** DHIS2, Outpatient, Inpatient, HMIS

## Abstract

**Background:**

Untimely, incomplete and inaccurate data are common challenges in planning, monitoring and evaluation of health sector performance, and health service delivery in many sub-Saharan African settings. We document Uganda’s experience in strengthening routine health data reporting through the roll-out of the District Health Management Information Software System version 2 (DHIS2).

**Methods:**

DHIS2 was adopted at the national level in January 2011. The system was initially piloted in 4 districts, before it was rolled out to all the 112 districts by July 2012. As part of the roll-out process, 35 training workshops targeting 972 users were conducted throughout the country. Those trained included Records Assistants (168, 17.3%), District Health Officers (112, 11.5%), Health Management Information System Focal Persons (HMIS-FPs) (112, 11.5%), District Biostatisticians (107, 11%) and other health workers (473, 48.7%). To assess improvements in health reporting, we compared data on completeness and timeliness of outpatient and inpatient reporting for the period before (2011/12) and after (2012/13) the introduction of DHIS2. We reviewed data on the reporting of selected health service coverage indicators as a proxy for improved health reporting, and documented implementation challenges and lessons learned during the DHIS2 roll-out process.

**Results:**

Completeness of outpatient reporting increased from 36.3% in 2011/12 to 85.3% in 2012/13 while timeliness of outpatient reporting increased from 22.4% to 77.6%. Similarly, completeness of inpatient reporting increased from 20.6% to 57.9% while timeliness of inpatient reporting increased from 22.5% to 75.6%. There was increased reporting on selected health coverage indicators (e.g. the reporting of one-year old children who were immunized with three doses of pentavelent vaccine increased from 57% in 2011/12 to 87% in 2012/13). Implementation challenges included limited access to computers and internet (34%), inadequate technical support (23%) and limited worker force (18%).

**Conclusion:**

Implementation of DHIS2 resulted in improved timeliness and completeness in reporting of routine outpatient, inpatient and health service usage data from the district to the national level. Continued onsite support supervision and mentorship and additional system/infrastructure enhancements, including internet connectivity, are needed to further enhance the performance of DHIS2.

## Background

An improved and harmonized health reporting system is critical for health system strengthening since it can generate timely information for proper planning, monitoring and evaluation of service delivery at all levels of the health system [1]. However, in most developing countries, particularly in sub-Saharan Africa, health reporting has been dominated by paper-based data collection and storage systems that tend to generate incomplete and inaccurate reports [2-4]. Evidence shows that the continued use of paper-based systems contributes to poor data quality in terms of reliability, availability, timeliness and completeness of reporting, and compromises health service delivery [2-4]. In Malawi, for instance, Makombe et al. [3] found that the use of paper-based health facility reports to generate national summaries resulted in a 12% underreporting of persons on first-line antiretroviral treatment because many sites did not submit accurate data to the national level. In South Africa, Garrib et al. [4] found that 2.5% of the total data values that should have been collected at 10 primary health care clinics using a paper-based system were missing while 25% of the data were outside the minimum and maximum values specified for the facilities. These findings call for a need to deploy web-based health management information systems in order to minimize errors in health reports and improve precision and usability of health data.

The development of web-based health information systems opened a new chapter for improving health reporting in the developed world and this is slowly taking root in most developing countries [4-6]. Web-based systems have facilitated the ability to collect more accurate and efficient data capture needed to inform planning and decision-making. For instance, in a study of the impact of a web-based reporting system on the collection of medication error occurrence data in the US, Rudman et al. [7] found that the introduction of a web-based software application increased the overall number of reported medication errors; the number of intercepted medication errors; and improved the documentation of number of physician-attributed medication errors. In South Africa, the implementation of a web-based data quality intervention improved data completeness from 26% before to 64% after the intervention; and improved overall accuracy of data from 37% at the first data audit to 65% at the third data audit [8]. These findings suggest that use of a web-based system can improve reporting consistency and data quality.

Uganda designed its first health information system (HIS) in 1985 to capture and analyze routine data on communicable and non-communicable diseases [9]. The system was specific and targeted certain disease conditions but later became obsolete due to the increasing need for collection of more relevant data. In 1992, a first revision of the HIS was done with the aim of capturing management information such as human and financial resources, drug and medical equipment, and routine disease data for further analysis. This revised version was piloted in two districts of Uganda before a national rollout was done in January 1997 [10]. In 2000, another review of the HIS was done which resulted into an expanded Health Management Information System (HMIS) that was able to capture data on more indicators, including indicators on vital management information that were not originally captured in the HIS, in order to guide planning and monitoring and evaluation of the health sector performance at national level. The review of the HIS was conducted in wide consultation with different stakeholders; development partners, local governments and non-government organizations (NGOs) committed to the implementation of Uganda’s Health Sector Strategic Plan (HSSP).

In 2007, with support from the Aid Capacity Enhancement (ACE) project, the Ministry of Health (MoH) Resource Centre – the centre responsible for managing health information within MoH – developed a more versatile, web-enabled platform which was piloted in 14 districts. However, by 2009, only a limited number of districts had adopted the web-enabled software, and a number of challenges still existed. These challenges included inability to produce reports, poor and unevenly developed infrastructure; poor capacity in data management by the health workforce, as well as uncoordinated collection and use of health information. In addition, fragmented data collection practices and silos of the HIS, inconsistent data quality and validity together with inadequate allocation of resources continued to affect the data collection process.

Thus, between 2000 and 2010, the HMIS continued to rely on paper-based forms that were used to collect data at community and health facility levels. These forms were summarized and dispatched to the district and then submitted to the national office (MoH) using various means including: electronic mail (e-mail), phone text messaging (SMS), faxing, and physical delivery. Data management at health facilities and districts was managed by Records Assistants, majority of whom were high school leavers without any basic skills in records data management [10,11]. This skills deficiency in data management contributed to the poor quality of data collected due to delayed reports, as well as incomplete and inaccurate submission of monthly routine reports to districts and later to national level. MoH introduced the district health management information software system version 2 (DHIS2) to further strengthen district-based health reporting. The aim of this paper is to document Uganda’s experience during the implementation and roll-out of the DHIS2 in strengthening district-based reporting in order to understand the lessons learnt and address the challenges encountered during the implementation process.

## Methods

### Introduction of DHIS2

The initial phase of the DHIS2 started in August 2010 when the U.S. Centers for Disease Control and Prevention (CDC) contacted the University of Oslo in a bid to outsource the electronic HMIS (DHIS2) for Uganda. The University of Oslo then customized the DHIS2 for Uganda, and in January 2011, Uganda adopted the electronic HMIS. Six months later, a technical team comprising staff with background training in information technology, public health, statistics, and monitoring and evaluation attended the DHIS2 training at the East Africa Academy in Dar es Salaam, Tanzania. During the training, the team documented lessons from other countries that had already initiated implementation of DHIS2 to inform its implementation in Uganda. Some of the lessons included the need for: 1) setting up the server environment and customization of the system, 2) piloting the customized system in some districts and performing a national roll-out in a phased manner, 3) training of trainers (ToTs), 4) training of district personnel prior to roll-out, 5) importing data from formerly used systems, and 6) piloting the use of mobile phones in data collection using the DHIS2 [7]. Full-scale implementation of the DHIS2 began in January 2012.

### DHIS2 customization and setup

Prior to full-scale implementation of DHIS2, there was a need to customize it to suit the Ugandan environment. This was done by a joint technical team composed of representatives from MoH (some of whom had attended the East Africa Academy training) and the Health Information System Programme (HISP) of the University of Oslo. The customization process took a period of four months and involved: definition of data elements, data sets, dash boards, and designing of data entry forms. The team also generated validation rules to ensure accurate entry of records into the system. Examples of validation rules that were developed included: (i) a rule on the number of pregnant mothers attending antenatal care for the fourth visit which was coded as ‘less than or equal to the number of those who attended the first antenatal visit’ (ANC4 < =ANC1), (ii) a rule on the number of pregnant mothers receiving the second dose of Intermittent Preventive Therapy (IPT2) which was coded as ‘less than or equal to the number of those who received the first Intermittent Preventive Therapy (IPT1)’ (IPT2 < = IPT1); and (iii) a rule that the number of people receiving the third dosage of diphtheria, pertussis and tetanus which was coded ‘less than or equal to the number that received the first dosage’ (DPT3 < = DPT1). In 2011, geographical information system mapping was done for purposes of mapping health facilities and this was customized into the system over a period of four months. On the other hand, HMIS tools for reporting such as outpatient & inpatient reporting forms were developed and customized into the system over the same period.

DHIS2 was initially installed on a testing server for training purposes and later migrated to the production server hosted at MoH central server room. This was aimed at improving ownership and provision of centralized technical support at a single point of control. Districts can access DHIS2 via the internet in the server–client architecture. In anticipation of information and communications technology infrastructural and internet connectivity challenges, the system was set to work in a hybrid mode. With this mode, users can continue to access the system and input data even when there is loss of internet connectivity. Data entered into the system in a hybrid mode can then be uploaded into the main system once internet connectivity is reinstated.

### Piloting the system

A few months after the technical team attended the DHIS2 East Africa Academy in Tanzania, MoH took the decision to roll-out DHIS2 to all districts. Training sessions were carried out in four pilot districts in Western Uganda (Kabarole, Kibaale, Kamwenge and Kyenjojo) in January 2012. The selection of the districts was informed by the presence of a project known as Saving Mothers Giving Life (SMGL). The SMGL project had already instituted a demand-driven mechanism for data collection at district level. The MoH then considered it prudent to pilot the system in the districts where the demand for data had been identified, as this would result in a rapid assessment of the performance of the system. The pilot phase was funded by the University of Oslo, but a few months later the MoH received additional funding from a PEPFAR/USAID project known as Monitoring Emergency Plan Progress (MEEPP) which supported the training of district-based staff in PEPFAR-funded districts.

### National roll-out and training approach

In preparation for the national roll-out, the country was zoned into 12 sub-regions to ensure that all the health facilities identified were captured in at least one of the sub-regions, so that the sizes of the classes per regional training were manageable. Two people responsible for data management were drawn from each of the 13 regional referral hospitals, 39 general hospitals, and 164 Health Center IV facilities to attend the training. In addition, one person responsible for data management was selected for training from each of the 803 Health Center III facilities. Data managers at all the health facilities were trained using the revised paper-based HMIS, while District Health Officers, District Biostatisticians and HMIS focal persons at districts or health sub-districts were trained on the electronic HMIS (DHIS2), together with staff from district-based implementing partners, surveillance officers, and monitoring and evaluation specialists.

A total of 35 training workshops were organized across all the 112 districts, and each workshop lasted for 5 working days running from 8:00 am-5:00 PM. In total, 972 users were trained. Of these, 168 (17.3%) were Records Assistants, 112 (11.5%) were District Health Officers, 112 (11.5%) were HMIS-Focal Persons (HMIS-FPs), 107 (11%) were District Biostatisticians, while majority (473, 48.7%) included other categories of health workers. All trainings were facilitated by officials from MoH in collaboration with CDC-Uganda and the rapid roll-out was organized with concerted efforts involving different implementing partners. However, by March 2014, the system had been rolled-out up to district level, with the lower level health units continuing to report using the paper-based system.

### Infrastructural enhancements

During the process of rolling out the system to the districts, 53 districts received a donation of computers and their accessories from the Uganda Communications Commission (UCC) through its Rural Community Development Fund project to support effective utilization of the system. The donation included fully networked computers that were connected to the internet with a free annual subscription for each district. In addition to the support from UCC, MoH, with support from CDC, set aside a budget to facilitate monthly renewal of internet access and routine technical support supervision at the districts.

### Importing data

In order to import data that were available before the installation of DHIS2, we imported data for 2011/12 into the DHIS2 system using the DHIS2 import function. These data already existed in management software databases such as EpiInfo, web-enabled databases, and Microsoft Excel.

### Indicator data extraction from DHIS2

In order to assess the progress made since the inception of DHIS2, we extracted outpatient (OPD), inpatient (IPD) and several health service coverage indicator data from DHIS2 for all the 112 districts during the period FY2012/13 and compared them with data imported into DHIS2 from databases that existed prior to the installation of DHIS2. Health service coverage indicators whose data were extracted included proportions of: pregnant women attending 4 antenatal care (ANC) sessions, pregnant women delivering in health facilities, one-year old children immunized against measles, pregnant women who completed intermittent preventive therapy (IPT2), human immunodeficiency virus (HIV)-exposed children accessing HIV testing services, and children under one year who were immunized with the third dose of pentavalent vaccine. We extracted data on two indicators of data quality, namely: completeness and timelines. We defined completeness as the proportion of health facility reports submitted to the district divided by the total number expected from the same district while timeliness in reporting was defined as the proportion of reports submitted by the deadline divided by actual reports received [9,10]. Service usage reporting rates were obtained and compared against MoH’s 2010–2015 Health Sector Strategic Investment Plan (HSSIP) targets. We also reviewed workshop training reports to document challenges encountered in the DHIS2 implementation.

## Results

There has been a remarkable improvement in both completeness and timeliness of data reporting after the installation of DHIS2. For instance, the proportion of districts submitting all reports to MoH (i.e. 100% reporting) increased from 2% to 20% for IPD and from 2.7% to 23.2% for OPD between 2011/12 and 2012/13. Similarly, the proportion of districts reporting below 50% declined from 75% to 21% for IPD and from 70% to 1% for OPD during the same period (Table 1).

**Table 1:** Inpatient (IPD) and outpatient (OPD) reporting rates FY 2011/12 and FY 2012/13

**Number of districts, n = 112**							
**District reporting rates (Proportion of districts reporting within the ranges)**							
**IPD reporting rates FY 2011/12 and FY 2012/13**							
	100%	90%-99%	80%-89%	70%-79%	60%-69%	50%-59%	<50%
2011/12	2%	1%	6.3%	3.6%	4.5%	8%	75%
2012/13	20%	22.3%	14.3%	13.4%	5.3%	4.5%	21%
**OPD reporting rates FY 2011/12 and FY 2012/13**							
	100%	90%-99%	80%-89%	70%-79%	60%-69%	50%-59%	<50%
2011/12	2.7%	10.7%	1.8%	6.3%	2.7%	6.3%	70%
2012/13	23.2%	52.7%	14.3%	6.3%	1.8%	1%	1%

**Figure 1:**
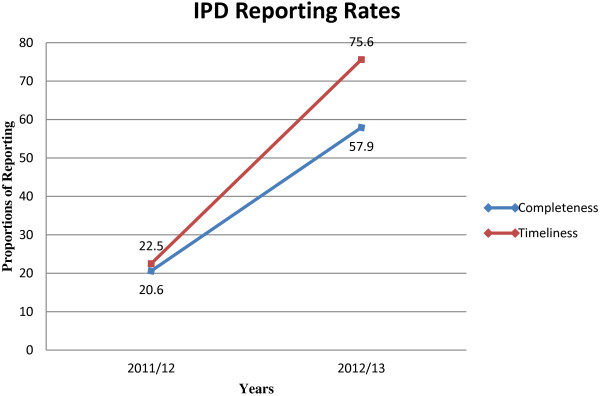
Inpatient department completeness and timeliness reporting for FY 2011/12 and FY 2012/13.

**Figure 2:**
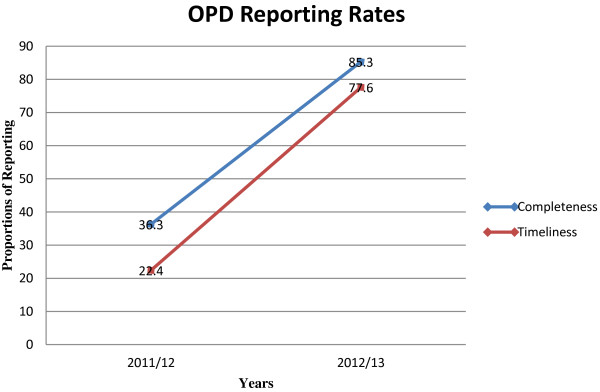
Outpatient department completeness and timeliness reporting for FY 2011/12 and FY 2012/13.

As noted in Table 2, regional IPD and OPD completeness and timeliness of reporting during 2011/12 was below 50% in all the regions for both indicators. However, there was a slight improvement observed during the 2012/13 for IPD completeness in reporting to between 54.9% and 61.5%. IPD timeliness in reporting was above 70% for all the regions. OPD completeness and timeliness in reporting improved in 2012/13 and was above 70% in all the regions. As a result, there was a corresponding improvement in national-level OPD and IPD reporting rates between 2012/13 and 2011/12 (Table 2). For instance, national-level completeness of inpatient forms increased from 20.6% to 57.9% while timely submission of the forms improved from 22.5% to 75.6% (Figure 1). Similarly, national-level completeness of outpatient forms increased from 36.3% to 85.3% while timeliness improved from 22.4% to 77.6% during this period (Figure 2).

**Table 2:** Completeness and timeliness of inpatient and outpatient reporting between FY 2011/12 and FY 2012/13 by region

	**Inpatient (IPD) reporting: 2011/12**					**Inpatient (IPD) reporting: 2012/13**				
	**Submitted reports (Actual reports)**	**Expected reports**	**% of expected reports (Completeness)**	**Submitted reports on time**	**% of submitted reports (Timeliness)**	**Submitted reports (Actual reports)**	**Expected reports**	**% of expected reports (Completeness)**	**Submitted reports on time**	**% of Submitted reports on time (Timeliness)**
Western	1249	5484	22.8	310	24.8	3183	5484	58	2366	74.3
Northern	1067	4836	22.1	107	10	2656	4836	54.9	2034	76.6
Eastern	689	3696	18.6	101	14.7	2273	3696	61.5	1608	70.7
Central	937	5160	18.2	367	39.2	2982	5160	57.8	2379	80.0
**National Level**	**3942**	**19176**	**20.6**	**885**	**22.5**	**11094**	**19176**	**57.9**	**8387**	**75.6**
	**Outpatient (OPD) reporting: 2011/12**					**Outpatient (OPD) reporting: 2012/13**				
	**Submitted reports (Actual reports)**	**Expected reports**	**% of expected reports (Completeness)**	**Submitted reports on time**	**% of submitted reports (Timeliness)**	**Submitted reports (Actual reports)**	**Expected reports**	**% of expected reports (Completeness)**	**Submitted reports on time**	**% of Submitted reports on time (Timeliness)**
Western	6272	14340	43.7	1642	26.2	12631	14340	88.1	9733	77.1
Northern	3642	9204	39.6	306	8.4	8027	9204	87.2	6329	78.8
Eastern	3458	11772	29.4	677	19.6	10411	11772	88.4	7723	74.2
Central	4404	13608	32.4	1381	31.4	10672	13608	78.4	8589	80.5
**National Level**	**17776**	**48924**	**36.3**	**4006**	**22.4**	**41741**	**48924**	**85.3**	**32374**	**77.6**

**Figure 3:**
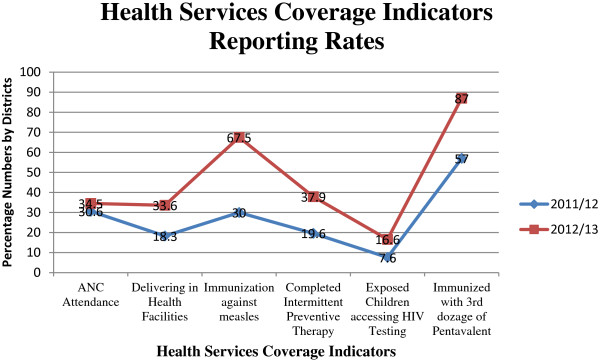
Health services usage reporting levels FY 2011/12 and FY 2012/13.

In addition to improving completeness and timeliness of IPD and OPD reporting, the implementation of DHIS2 has improved the reporting of health service coverage indicators. At the national level, reporting on the proportion of pregnant women attending four antenatal care (ANC) visits increased from 30.6% to 34.5% while reporting on the proportion of pregnant women delivering in health facilities increased from 18.3% to 33.6%. Similarly, reporting on the proportion of one-year old children immunized against measles increased from 30% to 67.5%; that of pregnant women who completed intermittent preventive therapy (IPT2) increased from 19.6% to 37.9% while that for the HIV-exposed children accessing HIV testing services within 12 months increased from 7.6% to16.6%. Over the same period, reporting on the proportion of children under one year who were immunized with the third dose of pentavalent vaccine increased from 57% to 87% (Figure 3).

### Implementation challenges

The greatest challenge during the DHIS2 roll-out was the limited technical staff at district level who can train as well as offer technical support supervision to health workers during the roll-out phase. In one case, for example, while the training was meant to last for 10 days, it was cut back to 5 days due to resource constraints, including lack of technical staff to conduct the training. As a result, the roll-out process took a much shorter period (i.e. six months) than the anticipated 12 months’ period, and this affected users who were computer illiterate and needed enough time to transition from the paper format to a computerized system.

In addition, there were problems of staff turn-over and redeployments that were a common occurrence at district level. This resulted in stalled use of DHIS2 in some districts due to lack of adequate trained staff to man the system and submission of incomplete forms and inaccurate data to the district health department. While this problem could be minimized through ongoing staff training at district level, this is likely to continue to persist, although plans are underway to secure funds to support the training of a pool of trainers that can offer the necessary support when new staffs are recruited or deployed at the district and facility levels.

Even after installation of DHIS2, errors in data collection continue to occur, and these errors are largely attributed to limited availability of reporting tools; lack of computers and poor access to the internet, lack of training of newly deployed or recruited staff and forgetfulness by trained users to capture relevant data. To address these challenges, there is a need to scale-up DHIS2 to lower-level health facilities to improve the quality of data collected at that level before they are submitted to the district and national levels.

Other challenges encountered included interrupted electricity supply to computers and lack of qualified staff to operate the computers. Also, the problem of regular power blackouts at MoH hindered reliable access to the central server room leading to unavailability of both the production and training server, and this resulted in intermittent use of the system even at MoH level.

## Discussion

Our experience shows that the installation and roll-out of DHIS2 to all districts in Uganda improved the timeliness and completeness of inpatient, outpatient reporting as well as reporting of selected health service coverage data from the district to the national (MoH) level. This increase in health reporting is likely due to the fact that health workers at the district level received training in how to input data into DHIS2, minimizing the errors and reducing bureaucratic delays that are associated with paper-based systems. With DHIS2, all primary data received from lower-level health facilities in paper form are immediately captured into DHIS2 at district level before they are sent to MoH. To support this process, the MoH Resource Centre has instituted routine support supervision and mentorship visits to all districts to strengthen the use of DHIS2 although challenges of limited resources in terms of personnel and funds continue to slow down the process.

There is evidence that the use of DHIS2 has improved health reporting in other countries, including Kenya, South Africa, and Malawi. For instance, both Kenya and South Africa reported improved reporting rates and effective tracking of health indicators following the adoption of DHIS2 [6,11]. In Malawi, a mid-term review of the performance of the district health information system judged it to be one of the best in Africa [5]. In Uganda, results from an earlier study by Kintu et al. [10] highlighted the numerous challenges that were inherent with the paper-based HMIS system. Some of these challenges seem to have been overcome through the use of the online health management information system.

Despite this level of success, the DHIS2 system has only been rolled down to the district level, with the lower-level health units continuing to report using the paper-based system. Our experience shows that paper-based forms from the lower-level health facilities continue to be submitted with inaccurate records, and this affects the quality of reports submitted to the districts and eventually to MoH. In the meantime, continued training of HMIS focal persons and routine data validation of paper-based reports can help to minimize these inaccuracies. However in the long-term, plans are necessary to scale-up the use of DHIS2 to all levels beyond the district in order to address other aspects of data accuracy.

### Lessons learned

The implementation of DHIS2 presented a number of lessons for future implementation of similar systems in Uganda or elsewhere. For instance, in order to address the challenge of limited availability of reporting forms, there is a need to distribute the HMIS tools to all health facilities ahead of time to minimize possibilities of delayed reporting. We learnt that the benefits associated with the installation of DHIS can better be maximized if lower-level health facilities collect accurate data and submit them on time to the district for entry into DHIS2. If the health facilities continue to submit incomplete or inaccurate data to the district level, even if they submitted them on time, there is a high possibility that the reports generated through DHIS2 would be equally inaccurate. This calls continuous support supervision to build health facility capacity to collect quality data to inform district and national level monitoring and planning [3].

We also learnt that in some cases, the installation of DHIS2 did not result in improved utilization of health reports generated at district level. Rather, districts focused on the need to submit complete and timely reports to MoH but with limited analysis of data to inform planning, decision-making and monitoring and evaluation of health service delivery at that level. Garrib et al. [4] have noted similar experiences in South Africa, suggesting that the culture of information use, especially at health facility and district levels, as opposed to the culture of reporting, is still weak in most settings, and is not improved through installation of DHIS2. To improve the utilization of DHIS2, there is a need for improved efforts to transform collected data into standard indicators that can be used to enhance health service delivery and quality of health care provided at district and lower-level health units.

### Limitations

The experiences shared are drawn from the process of rolling out the system since it was installed in 2012 rather than through outright primary data collection. This does not allow us to make definitive claims about the effect of implementing DHIS2 on data quality and reporting rates. In addition, the experience shared is based on a short duration of DHIS2 implementation (i.e. 1 year), suggesting that some of the challenges noted could have resulted from limited familiarity with the system. These are challenges that could eventually reduce with more time of implementation. To be able to obtain a more definitive picture of what it takes to implement a national-level online health management information system, we recommend that a comprehensive evaluation of the DHIS2 implementation be conducted after 3–4 years of implementation. This will enable sufficient documentation of successes and challenges encountered along the way. Such an evaluation should include an assessment of the cost-effectiveness of scaling-up the system from the national to the lower-level health facilities (beyond district-level) which could inform future planning and resource allocation.

## Conclusion

In summary, our experience shows that the roll-out and implementation of DHIS2 led to an improvement in timeliness and completeness in reporting for outpatient, inpatient and health service usage data from the district to the national (MoH) level. Continued onsite support supervision and mentorship and additional system/infrastructure enhancements may be required to further increase completeness and timeliness of the reports.

## Competing interests

The authors declare that they have no competing interests in the publication of this manuscript.

## Authors’ contribution

VK, JKBM and RKW conceptualized the need to write this paper, wrote the first draft of the paper; revised it for substantial intellectual content and were responsible for submission of the paper. CK and EM were responsible for the training and eventual roll-out of DHIS2 by virtue of their positions at the Ministry of Health Resource Center; and reviewed the draft paper and improved it for important intellectual content. FM contributed to the writing of the first draft of the paper, and reviewed it for substantial intellectual content. All authors read through and approved the paper for final submission.

## Pre-publication history

The pre-publication history for this paper can be accessed here:

http://www.biomedcentral.com/1472-6947/14/40/prepub

## References

[B1] Theo L Rainer S A framework for designing Health Information Systems 2000 Available at: http://libdoc.who.int/publications/2000/9241561998_(chp2).pdf. Accessed March 7, 2014

[B2] Ministry of Health, Health Systems 20/20, Makerere University School of Public Health Uganda Health Systems Assessment 2011 Kampala, Uganda and Bethesda, MD: Health Systems 20/20 Project, Abt Associates Inc Available at: http://health.go.ug/docs/hsa.pdf. Accessed March 6, 2014

[B3] Makombe SD Hochgesang M Jahn A Tweya H Hedt B Chuka S Yu JK Aberle-Grace J Pasulani O Bailey C Kamoto K Schouten EJ Harries AD Assessing the quality of data aggregated by antiretroviral treatment clinics in Malawi Bull World Health Organ 2008 86 310 314 10.2471/BLT.07.044685 18438520 PMC2647428

[B4] Garrib A Stoops N McKenzie N Dlamini L Govender T Rohde J Herbst K An evaluation of the District Health Information System in rural South Africa S Afr Med J 2008 98 549 552 18785397

[B5] Chaulagai CN Moyo CM Koot J Moyo HBM Sambakunsi TC Khunga FM Naphini PD Design and implementation of a health management information system in Malawi Health Policy Plan 2005 20 6 375 384 10.1093/heapol/czi044 16143590

[B6] Ayub MJB Lars Ø Ola D Jeremiah M Charles N National Roll out of District Health Information Software (DHIS 2) in Kenya IST-Africa 2012 Conference Proceedings

[B7] Rudman WJ Bailey JH Hope C Garrett P Brown CA Henriksen K, Battles JB, Marks ES The impact of a web-based reporting system on the collection of medication error occurrence data Advances in Patient Safety: From research to implementation 2005 Rockville, MD: Agency for Healthcare Research & Quality (US) 21249994

[B8] Mphatswe W Mate KS Bennett B Ngidi H Reddy J Barker PM Rollins N Improving public health information: a data quality intervention in Kwazulu-Natal, South Africa Bull World Health Organ 2012 90 176 182 10.2471/BLT.11.092759 22461712 PMC3314204

[B9] Mandelli A Giusti D Utilising The Health Management Information System (HMIS) For Monitoring Performance And Planning: Uganda Catholic Medical Bureau Experience Available at: http://www.ucmb.co.ug/data%20on%20ucmb/Reports/ARTICLES/Use%20of%20HMIS%20-%20UCMB%20experience.pdf. Accessed March 7, 2014

[B10] Kintu P Nanyunja M Nzabanita A Magoola R Development of HMIS in poor countries: Uganda as a case study Health Policy Dev 2005 3 1 46 53

[B11] Makumbi FE Okello C Mutebi A Wabwire-Mangen F Health Sector HIV/AIDs Response Review 2010 Kampala: Uganda Available at: http://danstan.me/HIVHSRReports/HIV%20HSR%20Final%20Reports(2)/HMIS%20Building%20Block%20Final%20Report%20Nov_2010.doc. Accessed March 7, 2014

